# Acute Necrotizing Pancreatitis Due to Hypertriglyceridemia: A Case Report

**DOI:** 10.7759/cureus.106469

**Published:** 2026-04-05

**Authors:** Pranaya Gade, Samantha Walkow, Marina Bastawross, Ayesha Ain, Antonios Tsompanidis

**Affiliations:** 1 Internal Medicine, Rowan School of Osteopathic Medicine, Stratford, USA; 2 Family Medicine, Hudson Regional Health-Secaucus University Hospital, Secaucus, USA

**Keywords:** acute pancreatitis, hypertriglyceridemia, intravenous insulin, necrotizing pancreatitis, severe pancreatitis

## Abstract

Necrotizing pancreatitis (NP) is a severe and potentially life-threatening complication of acute pancreatitis (AP). This case report discusses NP in a 39-year-old male patient with severe hypertriglyceridemia (HTG), highlighting management and treatment with supportive measures, intravenous insulin, antibiotic use, and total parenteral nutrition (TPN). Despite episodes of hypoglycemia requiring discontinuation of insulin, his triglyceride (TG) levels steadily declined without the need for plasmapheresis or surgical intervention. However, the pancreatitis worsened, leading to circulatory shock due to the acute inflammatory effect of NP and acute renal failure. This case emphasizes the importance of timely diagnosis and multidisciplinary supportive care in the successful treatment of HTG-induced NP.

## Introduction

Acute pancreatitis (AP) is a common gastrointestinal condition that can resolve with supportive care or sometimes become a medical emergency with a high mortality rate of 30% [1]. Severe pancreatitis can develop in 20% of patients with AP, sometimes including necrosis in the late phases, a serious complication that can lead to rapid organ failure [1-3]. Necrotizing pancreatitis (NP) is often caused by gallstones, alcohol consumption, and hypertriglyceridemia (HTG), with HTG NP severity being higher compared to biliary or alcoholic pancreatitis [4]. NP is generally diagnosed with contrast-enhanced computed tomography (CECT) depicting nonenhancement of pancreatic parenchyma or peripancreatic tissue or both [1,2]. Further complications of NP include infection, hemorrhage, pseudoaneurysm, venous thrombosis, bowel obstruction, and disseminated necrosis, causing organ failure [5]. Therefore, early diagnosis and treatment are imperative in acute NP.

NP is a rare and complicated condition with a risk of sudden organ failure and death, and few reported cases of positive outcomes [3]. In this article, we present a case of a 39-year-old male patient with NP secondary to HTG, a serum triglyceride (TG) level of 4145 mg/dL, complicated by shock, and successfully treated with intravenous (IV) fluid, insulin, trials of total parenteral nutrition (TPN), and antibiotics.

## Case presentation

A 39-year-old Spanish-speaking Hispanic male patient with a past medical history of pancreatitis, attributed to HTG in 2024, chronic HTG, and epilepsy, presented to the emergency department with acute onset of severe, sharp, constant abdominal pain rated 10/10 in intensity. The pain was localized to the epigastric region, non-radiating, and associated with nausea and three episodes of nonbilious nonbloody vomiting that began earlier that morning. He reported similar symptoms in 2024 during his prior pancreatitis episode, which he was told was from his HTG. He denied recent dietary changes, alcohol or drug use, smoking history, or trauma. He reported no past surgical history and no allergies. The patient’s only medication was carbamazepine 200 mg twice a day, prescribed over eight years ago by a physician in Guatemala. He worked in construction and denied any known family history of pancreatitis.

Upon admission, his pulse was 63 beats/minute, his blood pressure was 116/75 mmHg, his respiratory rate was 18 breaths/min, and his temperature was 97.5 ℉. On abdominal examination, the abdomen was tender in the epigastric region with hyperactive bowel sounds and no evidence of ascites. There was no organomegaly of the spleen, liver, or kidney. Other systemic evaluations were unremarkable. The laboratory findings on different days of admission are shown in Table 1.

**Table 1:** The patient's lab values during hospital stay Laboratory results recorded throughout the hospital stay demonstrated elevated lipase and triglyceride that downtrended with treatment. White blood cell count decreased as infection and inflammation subsided.

Parameter	First day of admission (6/14)	Second day of admission (6/15)	Third day of admission	Fourth day of admission	Fifth day of admission	Sixth day of admission	Normal range
Blood count							
Hemoglobin	15.2	-	-	-			12.5-16.3 g/dL
Hematocrit	45.1%	45.6%	43.6%	36.3%	32.2%	31.1%	36.7-47.1%
White blood cell count	14.2	12.7	11	7.9	6.0	7.4	3.6-10.2 K/uL
Serum lipase	40,424	-	-	1049			23-300 U/L
Serum lactate	2.3	4.7	-	-	-	-	0.6-2.1 mmol/L
Triglyceride	4,154	1,369	850	478	343	240	0-149 mg/dL
Cholesterol	323	250	-	-	-	-	0-199 mg/dL
High-density lipoprotein	20	31	-	-	-	-	30-70 mg/dL
Serum electrolytes							
Sodium (Na+)	133	135	134	134	137	135	132-148 mmol/L
Potassium (K+)	3.4	3.4	3.4	3.8	3.2	3.0	3.6-5.2 mmol/L
Chloride (Cl-)	111	99	93	96	100	99	98-107 mmol/L
Bicarbonate (HCO3-)	24.0	20.1	-	-	-	-	21-28 mmol/L
Serum calcium	5.3	5.7	5.6	6.2	6.7	7.1	8.6-10.4 mg/dl
Serum albumin	3.8	3.3	3.1	3.1	3.1	3.1	3.5-5.0 g/dL
Serum glucose	132	207	355	255	196	186	75-110 mg/dL
Serum creatinine	1.6	4.3	2.7	1.6	0.9	0.7	0.8-1.5 mg/dL
Liver function test							
Serum bilirubin (total)	1.2	2.0	2.7	2.2	1.7	1.5	0.2-1.3 mg/dL
Alkaline phosphatase	124	85	77	65	57	53	38-126 U/L
Alanine aminotransferase /Serum glutamic pyruvic transaminase	33	35	272	228	134	87	21-72 U/L
Prothrombin time				12.6	12.3	13.1	9.4-12.5 seconds
International normalized ratio				1.1	1.1	1.1	

**Figure 1:**
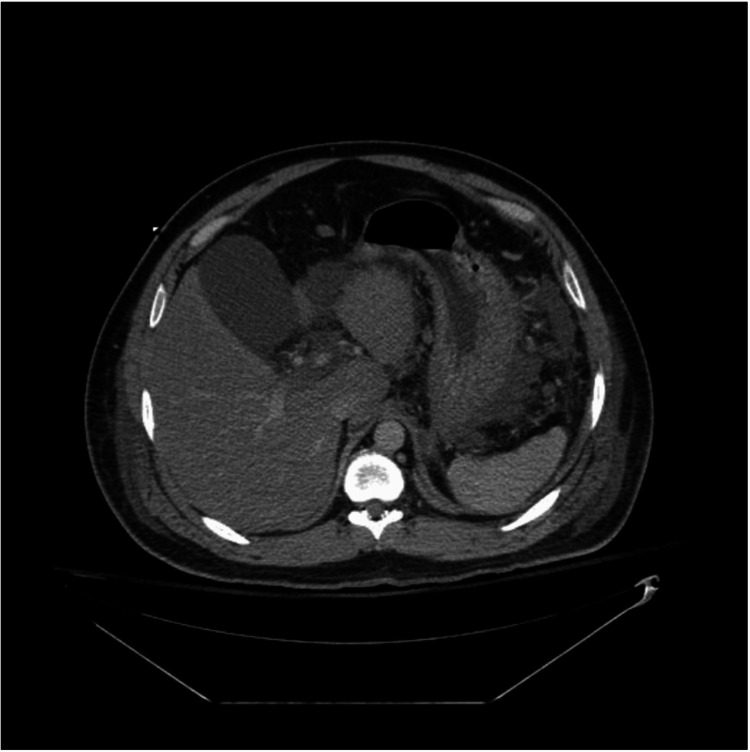
CT scan of the abdomen The CT scan showed interval progression of necrotizing pancreatitis within the pancreatic head, body, and tail with lobulated peripancreatic fluid collection, pancreatic parenchymal necrosis, possible focal hemorrhage within the pancreatic body and tail, and development of extensive peripancreatic, retroperitoneal edema.

A CT scan of the abdomen and pelvis without contrast, shown in Figure 1, revealed severe AP with swelling of the pancreatic head and surrounding inflammatory fluid, without evidence of pseudocyst or hemorrhage on the first day of admission.

The patient was admitted to the intensive care unit (ICU) for TG management with insulin infusion and for close monitoring of glucose levels while treated with insulin. He was kept nil per oral. To prevent hypoglycemia, IV fluids with dextrose were initiated. He received aggressive IV fluid resuscitation along with supportive therapies, including ketorolac, ondansetron, famotidine, vancomycin, piperacillin/tazobactam, and IV morphine and Dilaudid for pain control. Due to regular insulin treatments, the patient's electrolyte levels were closely monitored and appropriately treated with potassium chloride and calcium gluconate. His epilepsy was treated with Keppra. On the first night of admission, the patient developed metabolic acidosis and hypoxic respiratory failure requiring sodium bicarbonate, high-flow nasal cannula, and bilevel positive airway pressure (BiPAP) support. By the second day, he developed hypotension requiring norepinephrine, and labs indicated acute kidney injury, prompting nephrology consultation and increased fluid administration with a Foley catheter placement. Despite holding insulin due to hypoglycemia, his triglyceride levels continued to decline. Chest imaging over the following days showed evolving bilateral pleural effusions, pulmonary congestion, and right hemidiaphragm elevation. On hospital day three, the patient reported improving abdominal pain, insulin infusion was stopped, and urine output increased with the patient having 400 cc in his Foley bag over a 2.5-hour period. The WBC count increased to 14.1 K/µL. The patient was started on meropenem and vancomycin for coverage of intra-abdominal infection per recommendation from the infectious disease specialist. A repeated CT scan on the fourth day showed NP, and the patient was placed on TPN, meropenem, and fenofibrates. Surgery was consulted, and no intervention was determined. Supportive care was continued with opioids for pain control and lactated Ringer's solution for IV fluids as needed. After the patient was hemodynamically stable, he was downgraded to telemetry for further management. TG levels improved to 240 mg/dL. TPN was discontinued when the patient started tolerating liquid, then a low-fat diet. Eventually, the patient felt well and was discharged home on oral medication with advice from endocrinology, gastroenterology, and infectious disease. Per neurology recommendation, the patient was told to stop carbamazepine and take Keppra instead.

## Discussion

This case highlights the targeted management required for HTG-induced pancreatitis (HTGP) in a patient without a history of alcohol use, gallstones, or other common causes of NP. The prevalence of HTGP is around 22%, making HTG the third most common cause of AP and associating it with more severe complications and higher mortality rates [6]. Diagnosis of HTGP is indicated by a TG level > 1000 mg/dL, while this patient had a serum TG level of 4145 mg/dL [6]. The goal is to decrease the serum TG levels to <500 mg/dL before discharge [7]. Management for HTG NP that has been successful in the past includes IV fluid, insulin or heparin, plasmapheresis, and early enteral nutrition during the acute phase and lifestyle modifications and medication therapy with fenofibrate, due to its slow onset of action, for long-term management [7,8].

Multiple studies have shown a decrease in serum TG due to insulin therapy [9-11]. Insulin is a common initial treatment option for decreased serum TG levels, as insulin enhances the synthesis of lipoprotein lipase (LPL), which hydrolyzes TG [9]. Sometimes, if the patient responds well to insulin therapy, there is no need for consideration of plasmapheresis, as with this patient, whose TG levels decreased by 94.2% in six days, with over 50% (67%) decrease occurring by the second day of admission. Serum TG levels steadily decreased throughout the patient’s hospitalization from 4145 mg/dL to 240 mg/dL with consistent daily insulin treatment. This opens a discussion for further research exploring the connection between insulin deficiency and HTGP. Heparin treatment is being explored due to its ability to transiently stimulate the release of endothelial LPL and, therefore, help decrease serum TG levels [12].

Plasmapheresis can further lower excessively elevated TG levels and is most beneficial when started earlier in the clinical course [12]. However, plasmapheresis is a relatively expensive treatment, and a study has shown that more patients treated with plasmapheresis requiring ICU admission in comparison to patients that were medically managed [13]. This patient responded well to pharmacologic management and did not undergo plasmapheresis during the hospitalization, but other reports have indicated successful treatment with plasmapheresis in patients with persistently elevated TG levels [7,11].

Depending on the classification and severity of the NP, more invasive treatment measures can be considered. The four classifications for severe pancreatitis are acute peripancreatic fluid collection, pancreatic pseudocyst, acute necrotic pancreatic collections, and walled-off necrosis [14]. In the cases of a pancreatic pseudocyst, endoscopic ultrasound-guided fine-needle aspiration can be considered, while for walled-off necrosis, surgical drainage and debridement are to be considered [14]. Surgical intervention was not indicated for this patient, and management continued with pharmacologic treatment.

Supportive care with proper nutrition is important to maintain gastrointestinal motility, intestinal mucosal integrity, and splanchnic circulation [14]. Early oral feeding is associated with decreased mortality, but if not tolerated due to nausea or vomiting, enteral nutrition can be considered as an alternative [14,15]. TPN was used for this patient to achieve proper nutrition throughout the hospital stay. Early addition of broad-spectrum antibiotics to the treatment plan, if there is a suspected infection, is important to prevent progression to sepsis, as seen in this patient with the addition of vancomycin, metronidazole, Zosyn, and meropenem [15].

## Conclusions

NP is a severe complication of AP that requires prompt recognition and a multidisciplinary management approach to prevent life-threatening outcomes. Early recognition of pancreatitis caused by HTG is feasible in a clinical setting. This case report demonstrates early treatment of NP and associated complications, including acute kidney injury and shock, in a patient with NP secondary to an especially elevated serum TG level. Treatment with IV fluid and insulin can decrease serum TG levels significantly; early enteral nutrition for supportive care and long-term management with fenofibrate and lifestyle modification can help prevent recurrent HTG NP. These interventions helped avoid a more fatal prognosis associated with NP. Serial lipid panels to confirm adequate downtrending of serum TG in response to treatment are important. Newer treatments targeting apolipoprotein C3 are being developed and incorporated into HTG treatment. It is important to continue to investigate successful treatment options for HTG NP to prevent organ failure, given the variability in clinical presentation and clinical course.
